# Outcomes after gastrectomy according to the Gastrectomy Complications Consensus Group (GCCG) in the Dutch Upper GI Cancer Audit (DUCA)

**DOI:** 10.1007/s10120-024-01527-0

**Published:** 2024-06-28

**Authors:** Maurits R. Visser, Daan M. Voeten, Suzanne S. Gisbertz, Jelle. P. Ruurda, Mark I. van Berge Henegouwen, Richard van Hillegersberg, the Dutch Upper Gastrointestinal Cancer Audit (DUCA) Group

**Affiliations:** 1 Department of Surgery, University Medical Center Utrecht, University of Utrecht, Utrecht, The Netherlands; 2 Scientific Bureau, Dutch Institute for Clinical Auditing, Leiden, The Netherlands; 3 Department of Surgery, Amsterdam UMC Location Vrije Universiteit, Amsterdam, The Netherlands; 4 Cancer Treatment and Quality of Life, Cancer Center Amsterdam, Amsterdam, The Netherlands; 5 Department of Surgery, Amsterdam UMC Location University of Amsterdam, Amsterdam, The Netherlands

**Keywords:** Gastric carcinoma, Gastrectomy, Complications

## Abstract

**Background:**

In 2019, the Gastrectomy Complications Consensus Group (GCCG) published a standardized set of complications aiming toward uniform reporting of post-gastrectomy complications. This study aimed to report outcomes after gastrectomy in the Netherlands according to GCCG definitions and compare them to previously reported national results and the European database reported by the GCCG.

**Methods:**

This nationwide, population-based cohort study included all patients undergoing gastrectomy for gastric cancer registered in the DUCA in 2020–2021. Postoperative morbidity and 30-day/in-hospital mortality were analyzed according to the GCCG definitions. For all patients, baseline characteristics and outcomes were compared with the GCCG cohort consisting of 27 European expert centers (GASTRODATA; 2017–2018).

**Results:**

In 2020–2021, 782 patients underwent gastrectomy in the Netherlands. Variation was seen in baseline characteristics between the Dutch and the GCCG cohort (*N* = 1349), most notably in minimally invasive surgery (80.6% vs 19.6%, *p* < 0.001). In the Netherlands, 223 (28.5%) patients developed a total of 407 complications, the most frequent being non-surgical infections (28.5%) and anastomotic leakage (13.4%). The overall complication and 30-day mortality rates were similar between the Dutch and GCCG cohort (28.5% vs 29.8%, *p* = 0.563; 3.7% vs 3.6%, *p* = 0.953). Higher surgical and endoscopic/radiologic reintervention rates were observed in the Netherlands compared to the GCCG cohort (10.7% vs 7.8%, *p* = 0.025; 10.9% vs 2.9%, *p* < 0.001).

**Conclusion:**

Reporting outcomes according to the standardized GCCG definitions allows for international benchmarking. Postoperative outcomes were comparable between Dutch and GCCG cohorts, but both exceed the international benchmark for expert gastrectomy care, highlighting targets for national and international quality improvement.

**Supplementary Information:**

The online version contains supplementary material available at 10.1007/s10120-024-01527-0.

## Introduction

Gastric cancer is the fifth most common form of cancer, with a yearly incidence of over one million new patients, and the third leading cause of cancer death worldwide [1]. Potentially curative treatment usually consists of surgical resection combined with perioperative chemotherapy [2, 3]. Subtotal and total gastrectomy require specific expertise and are associated with substantial morbidity and even mortality. In Western countries, postoperative mortality varies between 1 and 7% [4, 5].

An even wider range exists for morbidity, with rates ranging from 11 to 46% [5, 6]. The Gastrectomy Complications Consensus Group (GCCG) suggests that a wide variation in definitions, terminology and recording of postoperative complications are partly attributable to these varying rates. To find common ground in reporting gastrectomy-related complications, the GCCG hosted multiple Delphi-rounds among upper-gastrointestinal surgery experts from different countries, following the example of the Esophagectomy Complications Consensus Group (ECCG) [6]. In 2019, the GCCG published a comprehensive list of surgery-related and gastric-cancer-specific complications, enabling accurate international comparisons of complications after gastrectomy [6]. Following this publication, the Dutch Upper Gastrointestinal Cancer Audit (DUCA) amended the complication registry towards GCCG definitions in 2020.

Similar standardized complication definitions for esophageal cancer surgery were published by the ECCG in 2015. The results of 24 high-volume European hospitals according to ECCG definitions were published in 2019 [7, 8]. After amending the DUCA to the ECCG definitions, a comparison between the DUCA and European cohorts yielded valuable information [9]. In the Netherlands, anastomotic leakage and pneumonia rates were significantly higher compared to the European cohort, even though similar definitions were used now. These differences resulted in the establishment of the first national best-practice meetings to improve nationwide anastomotic leakage rates after esophagectomy [9, 10].

After publication of the GCCG definitions, outcomes following gastrectomy of 27 European expert centers were published using data from the GASTRODATA database [11]. When comparing these data to previously reported Dutch results, differences are present in both mortality and complication rates between the Netherlands and the international database of the GCCG study [9, 11, 12]. However, the Dutch studies did not report complications according to GCCG definitions, possibly hindering an accurate comparison. The aim of this study was to report postoperative morbidity and mortality after gastrectomy in the Netherlands according to the definitions of the GCCG to enable accurate comparisons. Furthermore, we aimed to compare these results to (1) previously reported Dutch results that did not use uniform complication definitions to assess its impact on comparisons and (2) make an accurate comparison with the European database reported by the GCCG to identify potential areas for improvement.

## Methods

This nationwide, population-based cohort study retrieved data from the mandatory DUCA registration. Dutch hospitals are obligated to register all patients undergoing surgery for esophageal or gastric cancer with the intent of resection in this database. Postoperative complications are registered until 30 days after surgery or discharge. The DUCA was amended to the GCCG definitions in 2020, adding eight specific complications. The DUCA consists of 65 individual complications from both the GCCG and ECCG definitions, as well as other complications deemed relevant by Dutch surgeons. An overview of definitions in relation to GCCG definitions is shown in Supplementary Table S1. No informed consent or ethical review is required under Dutch law, as patient and hospital data are registered anonymously. The DUCA scientific committee reviewed and approved this study’s protocol (DUCA202105).

### Patients

All patients undergoing gastrectomy for gastric cancer registered in the DUCA from 1-1-2020 until 31-12-2021 were included. Patients with a palliative bypass procedure or non-resectable disease (locoregional or metastases) during intentional gastrectomy were excluded. Patients with missing data on postoperative complications and/or 30-day/in-hospital mortality were also excluded.

### Endpoints

The primary endpoint was 30-day/in-hospital mortality. Secondary endpoints were: postoperative outcomes according to the definitions of the GCCG, consisting of 27 perioperative complications, as well as the number and type of reinterventions, escalation in level of care (to ICU), number of hospital readmissions, postoperative hospitalization and number of days on ICU [6]. Patient, tumor and treatment characteristics and outcomes were compared with the European cohort of the GCCG study (GASTRODATA database [13]; 2017–2018), comprised of patients from 27 European expert centers, including four Dutch hospitals. Complications were reported as complication rates or as the proportion of total complications. Complications registered in the DUCA, but not in the GCCG definitions, were also described.

### Statistical analysis

Postoperative outcomes were described using frequencies and percentages. Complication severity was reported by the median Clavien–Dindo score and the total Comprehensive Complication Index (CCI) [14] score. Continuous variables were reported as median and range. Baseline characteristics and outcomes were compared between the DUCA and GCCG cohorts using Chi-square analyses. Two-sided *p* values < 0.05 were considered statistically significant. All statistical analyses were performed using R-studio version 1.4.1106, The R Foundation for Statistical Computing [15].

## Results

### Patients

Between January 2020 and December 2021, a total of 782 patients undergoing gastrectomy for gastric cancer were registered in the DUCA. One patient was excluded due to missing length of hospital stay data. Patient, tumor, and treatment characteristics of the DUCA and GCCG cohorts are shown in Table 1. The cohorts differed significantly on all baseline characteristics, except for sex and surgical procedure. In the DUCA cohort, 63.2% of patients underwent neo-adjuvant treatment compared to 45.1% in the GCCG cohort (*p* < 0.001). Minimally invasive surgery was performed in 80.6% of patients in the DUCA cohort, compared to 19.6% in the GCCG (*p* < 0.001).

**Table 1:** Patient, tumor and treatment characteristics of the DUCA and GCCG cohorts

	**DUCA**		**GCCG**^**A**^		*P* value (*χ*^2^)
	*N* (%)	Median [range]	*N* (%)	Median [range]	
Total	782 (100)	–	1349 (100)	–	
Patients per center	–	42 [1–116]	–	47.0 [23–171]	
*Sex*					0.437
Male	490 (62.7)	–	821 (60.9)	–	
Female	292 (37.3)	–	528 (39.1)	–	
Age in years		71.0 [21–93]	–	69.0 [19–93]	
*BMI*					
< 18.5	24 (3.1)	–	58 (4.3)	–	**0.008**
18.5–25	377 (48.2)	–	556 (41.2)	–	
25–30	272 (34.8)	–	411 (30.5)	–	
> 30	81 (10.4)	–	184 (13.6)	–	
Missing	28 (3.6)		140 (10.4)		
*ASA–score*					** < 0.001**
I	42 (5.4)	–	190 (14.1)	–	
II	390 (49.9)	–	629 (46.6)	–	
III	318 (40.7)	–	438 (32.5)	–	
IV	24 (3.1)	–	25 (1.9)	–	
Missing	8 (1.0)		67 (5.0)		
*Charlson comorbidity Index*					** < 0.001**
0	288 (36.8)	–	118 (8.7)	–	
1–4	445 (56.9)	–	888 (65.8)	–	
5–8	44 (5.6)	–	311 (23.1)	–	
9–13	5 (0.6)	–	32 (2.4)	–	
*Tumor location (multiple answers allowed)*					** < 0.001**
Fundus	51 (6.5)	–	228 (17.3)	–	
Corpus	260 (33.2)	–	358 (27.1)	–	
Antrum/pylorus	357 (45.7)	–	638 (48.3)	–	
Total Stomach	30 (3.8)	–	42 (3.2)	–	
Rest stomach/anastomosis	19 (2.4)	–	–	–	
Cardia/GEJ	57 (7.3)	–	169 (12.8)	–	
*Histology*					** < 0.001**
Adenocarcinoma	753 (96.3)	–	1,205 (90.9)	–	
Squamous cell carcinoma	3 (0.4)	–	2 (0.2)	–	
Other/unknown/missing	34 (4.4)	–	118 (8.9)	–	
*Clinical tumor stage*					** < 0.001**
T0-2	162 (20.7)	–	386 (28.6)	–	
T3–4	539 (68.9)	–	816 (60.5)	–	
Tx	81 (10.4)	–	147 (10.9)	–	
*Clinical node stage*					** < 0.001**
N0	429 (54.9)	–	481 (35.7)	–	
N +	322 (41.2)	–	643 (47.7)	–	
Nx	31 (4.0)	–	225 (16.7)	–	
*Clinical metastasis stage*					** < 0.001**
M0	712 (91.0)	–	906 (67.2)	–	
M +	27 (3.5)	–	110 (8.2)	–	
Mx	43 (5.5)	–	333 (24.7)	–	
*Neoadjuvant therapy*					** < 0.001**
Chemoradiotherapy	50 (6.4)	–	15 (1.1)	–	
Chemotherapy	444 (56.8)	–	577 (42.8)	–	
Radiotherapy	0 (0)	–	16 (1.2)	–	
None/missing	288 (36.8)	–	741 (54.9)	–	
*Surgical procedure*					**0.054**
Total gastrectomy	374 (47.8)	–	705 (52.2)	–	
Subtotal gastrectomy	408 (52.2)	–	641 (47.5)	–	
Missing	0 (0)		3 (0.2)		
*Surgical approach*					** < 0.001**
Open	152 (19.4)	–	1,081 (80.1)	–	
Minimally invasive	630 (80.6)	–	264 (19.6)	–	
Conversion to open (Yes)	43 (5.5)	–	29 (11.0)	–	
Missing	0 (0)		4 (0.3)		
*Resection margin*					**0.023**
R0	717 (91.7)	–	1,242 (92.1)	–	
R1	60 (7.7)	–	91 (6.7)	–	
R2	1 (0.1)	–	16 (1.2)	–	
Missing	4 (0.5)		0 (0)		
Number of resected lymph nodes	–	25.0 [0–82]	–	31.0 [0–127]	–
Number of positive lymph nodes	–	4.0 [0–65]	–	1.0 [0–57]	–

A. Baiocchi GL, Giacopuzzi S, Reim D, et al. Incidence and grading of complications after gastrectomy for cancer using the GASTRODATA registry. A european retrospective observational study. *Ann Surg* 2020.

### Overall outcomes according to GCCG

In the Netherlands, postoperative complications occurred in 223 (28.5%) patients, developing a total of 407 complications, compared to a complication rate of 29.8% (*p* = 0.563) in the GCCG cohort, consisting of 625 complications in 1349 patients (Table 2). In both cohorts, 30-day mortality rates were similar (3.7% vs 3.6%, *p* = 0.953). More complications occurred per patient in the Netherlands compared to the GCCG cohort (1.8 vs 1.5, respectively) and there was a significantly higher proportion of Clavien–Dindo grade II complications (37.3% vs 30.7%, *p* = 0.032) in the Netherlands. Also, more patients underwent surgical (10.7% vs 7.8%, *p* = 0.025) and endoscopic/radiologic (10.9% vs 2.9%, *p* < 0.001) reinterventions in the Netherlands. Reasons for reinterventions in the DUCA are presented in Table S2. Failure-to-rescue rates were similar (13.0% vs 11.9%, *p* = 0.794). The median in-hospital stay was higher in the GCCG cohort (6 vs 9 days). Only reported in the Netherlands, the median ICU stay was 0 days and in-hospital/30-day mortality rate was 4.1%.

**Table 2:** Overview postoperative outcomes in the DUCA and GCCG cohorts

Total patient episodes in DUCA = 782	DUCA		GCCG^A^	P va*l*ue (*χ*^2^/Fisher)
	Number	Percentage	Percentage (number)	
Patients developing at least 1 complication (all DUCA complications included)	265	33.9	–	–
Patients developing at least 1 complication^B^	223	28.5	29.8	0.563
*Clavien–Dindo grading of individual complications*				
Grade I	21	5.2	6.4	0.490
Grade II	152	37.3	30.7	**0.032**
Grade IIIa	79	19.4	21.6	0.442
Grade IIIb	76	18.7	19.0	0.948
Grade IVa	37	9.1	8.5	0.820
Grade IVb	13	3.2	3.7	0.809
Grade V	29	7.1	10.1	0.130
All	407	–	(625)	–
Complications per patient	1.8	–	(1.5)	–
Patients requiring surgical reinterventions	84	10.7	7.8	**0.025**
Patients requiring endoscopic and/or radiological interventions	85	10.9	2.9	** < 0.001**
Escalation in level of care (mostly to ICU)	51	6.5	6.2	0.859
*Discharge location*				
Home	712	91.0	91.9	0.537
Secondary medical facility/rehab	41	6.5	4.9	0.136
*Readmission within 30 days after discharge*				
Readmissions related to gastrectomy	69	8.8	7.1	0.125
Readmissions unrelated to gastrectomy	35	4.5	3.6	0.303
30-day mortality	29	3.7	3.6	0.953
In-hospital/30-day mortality	32	4.1	–	–
Failure to rescue	29/223	13.0	11.9	0.794

	DUCA		GCCG^A^	
	Median	Range	Median	Range
Comprehensive complications index (CCI)	33.5	(8.7–100)	26.2	(8.7–100
Postoperative hospitalization, days	6	(2–105)	9	(1–142)
Number of days on ICU	0	(0–47)	–	–

^A^Baiocchi GL, Giacopuzzi S, Reim D, et al. Incidence and grading of complications after gastrectomy for cancer using the GASTRODATA registry. A European retrospective observational study. *Ann Surg* 2020

^B^According to GCCG complications definitions: Baiocchi GL, Giacopuzzi S, Marrelli D, et al. International consensus on a complications list after gastrectomy for cancer. *Gastric Cancer* 2019

### Individual outcomes according to GCCG

Table 3 shows the GCCG definitions of individual complications. Unintended intraoperative damage to major vessels and/or organs had a proportion of all complications of 3.4% in the Netherlands, compared to 1.1% in the GCCG cohort (*p* = 0.019), although the. For postoperative general complications, non-surgical infections (27.5%) occurred most frequently in the Netherlands, similar to the GCCG cohort (23.0%). The proportion of pleural effusion requiring drainage was higher in the Netherlands compared to the GCCG cohort (4.7% vs 8.3%, *p* = 0.032), with similar proportions of the other general complications. In the Netherlands, anastomotic leakage (12.8%), other postoperative abnormal fluid or abdominal collections requiring treatment (9.1%), postoperative bowel obstruction (8.6%) and other complications requiring re-intervention or other invasive procedures (6.4%) were the most frequent postoperative surgical complications. Details on these four surgical postoperative complications, accounting for 150 (38.9%) complications, are shown in Table 4. Of the postoperative surgical complications, pancreatic fistula (1.2% vs 4.0%, *p* = 0.016) and pancreatitis (0% vs 1.9%, *p* = 0.012) had a lower proportion of all complications compared to the GCCG cohort, with a higher proportion of postoperative bowel obstruction (8.6% vs 4.8%, *p* = 0.020) in the Netherlands. All other proportions of postoperative surgical complications were similar between the cohorts.

**Table 3:** Incidence and grading of all specific GCCG complications in the DUCA and GCCG cohorts

	DUCA			GCCG^B^			*P*-value (χ^2^)
	Number of adverse events	Percentage of adverse events	Clavien–Dindo score (median)	Number of adverse events	Percentage of adverse events	Clavien–Dindo score (median)	
*Intraoperative*							
1. Unintended intraoperative damage to major vessels and/or organs requiring reconstruction or resection	14	3.4	–	7	1.1	–	**0.019**
2. Intraoperative bleeding requiring urgent treatment	9	2.2	–	6	0.9	–	0.169
3. Unexpected medical conditions interrupting or changing the planned procedure	–	–	–	0	0	–	–
*Postoperative general*							
4. Stroke causing patient’s permanent deficit	2	0.5	II	1	0.2	V	0.708
5. Need for CPR	2	0.5	IVb	9	1.4	V	0.254
6. Myocardial infarction	1	0.2	IVa	5	0.8	IIIa	0.468
7. Cardiac dysrhythmia requiring invasive treatment	2	0.2	IVa	2	0.3	IIIb	1.000
8. Acute myocardial failure with acute pulmonary edema	5	1.2	II	3	0.5	II	0.329
9. Pulmonary embolism	8	2.0	II	8	1.3	II	0.540
10. Respiratory failure requiring reintubation	11	2.7	IVa	34	5.4	IVa	0.051
11. Need for tracheostomy	1	0.2	–	9	1.4	IVa	0.112
12. Pleural effusion requiring drainage	19	4.7	IIIa	52	8.3	IIIa	**0.032**
13. Pneumothorax requiring treatment	1	0.2	IIIa	7	1.1	IIIa	0.229
14. Need for prolonged intubation (> 24 h after surgery)	4	1.0	–	16	2.6	II	0.118
15. Acute liver dysfunction (Child–Pugh score > 8 for 48 + hours)	3	0.7	II	1	0.2	I	0.344
16. Acute renal insufficiency/renal failure requiring CVVH or dialysis	4	1.0	II	18	2.9	IIIa	0.066
17. Non-surgical infections^C^	112	27.5	II	144	23.0	II	0.120
*Postoperative surgical*							
18. Postoperative bleeding requiring invasive treatment	24	5.9	IIIb	35	5.6	IIIb	0.949
19. Postoperative bowel obstruction	35	8.6	II	30	4.8	II	**0.020**
20. Postoperative bowel perforation or necrosis	8	2.0	IIIb	11	1.8	IVa	0.997
21. Duodenal leak	11	2.7	IIIb	22	3.5	IIIb	0.584
22. Anastomotic leak	52	12.8	IIIa	61	9.8	IIIb	0.157
23. Postoperative pancreatic fistula	5	1.2	IIIa	25	4.0	II	**0.016**
24. Postoperative pancreatitis	0	–	–	12	1.9	II	**0.012**
25. Other postoperative abnormal fluid from drainage and/or abdominal collections without gastrointestinal leak(s) preventing drainage removal and/or requiring treatment	37	9.1	IIIa	58	9.3	IIIa	1
26. Delayed gastric emptying (by 10th postoperative day)	11	2.7	IIIa	14	2.2	IIIa	0.791
27. Other major complications requiring re-intervention or other invasive procedures^D^	26	6.4	IIIb	35	5.6	IIIb	0.697
Total	407			625			

^A^Complication definititions according to the GCCG definitions: Baiocchi GL, Giacopuzzi S, Marrelli D, et al. International consensus on a complications list after gastrectomy for cancer. *Gastric Cancer* 2019

^B^Baiocchi GL, Giacopuzzi S, Reim D, et al. Incidence and grading of complications after gastrectomy for cancer using the GASTRODATA registry. A european retrospective observational study. *Ann Surg* 2020

^C^Includes gastrointestinal, respiratory, renal / urinary and other infections

^D^Includes evisceration, diaphragmatic hernia, feeding jejunostomy-related complications and other reoperations not caused by postoperative bleeding, leakage or bowel necrosis

**Table 4:** Characteristics of the four most frequent surgical complications in the DUCA and GCCG cohorts

	No. in DUCA	Percentage in DUCA	Percentage in GCCG^A^	*P*-value (*χ*^2^/Fisher)
Patients with anastomotic leak	52	6.6	4.5	**0.044**
Grade I–II	5	9.6	16.4	0.435
Grade IIIa–IVb	43	82.7	67.2	0.097
Grade V	4	7.7	16.4	0.266
*Anastomosis*				
Esophagojejuno	47	90.4	89.3	1
Gastroentero	4	7.7	8.9	1
Entero-entero	1	1.9	1.8	1
*Surgical procedure*				
Total gastrectomy	47	90.4	83.6	0.435
Subtotal gastrectomy	5	9.6	16.3	0.435
Anastomotic leakage rate total gastrectomy	47/374	12.6	7.2	**0.005**
Anastomotic leakage rate subtotal gastrectomy	5/408	1.2	1.5	0.856
Patients requiring reinterventions (multiple options allowed)	41	78.8	68.9	0.325
Surgical	27	51.9	42.6	0.425
Endoscopic	32	61.5	27.9	** < 0.001**
Radiologic	11	21.2	16.4	0.685
Escalation in level of care (ICU)	19	36.5	–	–
Failure to cure	4/52	7.5	16.4	0.266
Deceased patients having this complication	10	1.3	1.5	0.846
Postoperative hospitalization, days (median, range)	19 (2–105)	–	32.0 (1–100)	–
CCI (median, range)	41.6 (20.9–100)	–	45.2 (20.9–100)	–

	No. in DUCA	Percentage in DUCA	Percentage in GCCG^A^	*P*-value (χ^2^/Fisher)
Patients with other postoperative abnormal fluid requiring treatment	37	4.7	4.3	0.721
Other drainage of abnormal fluid collections	12	27.9	69.0	** < 0.001**
Chyle leak	9	20.9	24.1	0.889
Intra-thoracic / intra-abdominal abscess	22	51.2	–	–
*Severity score*				
Grade I–II	14	32.6	48.3	0.167
Grade IIIa–IVb	28	65.1	50.0	0.190
Grade V	1	2.3	1.7	1
*Surgical procedure*				
Total gastrectomy	25	67.6	67.3	1
Subtotal gastrectomy	12	32.4	32.7	1
Patient requiring reinterventions (multiple options allowed)	28	75.7	31.0	** < 0.001**
Surgical	13	35.1	13.7	**0.028**
Endoscopic	8	21.6	3.4	**0.013**
Radiologic	20	54.1	39.6	0.245
Escalation in level of care	8	21.6	10.3	0.224
Failure to cure	1/37	2.7	1.7	1
Deceased patients with this complication	2	0.3	0.2	1
Postoperative hospitalization, days (median, range)	19 (4–89)	–	20 (7–120)	–
CCI (median, range)	33.7 (8.7–100)	–	26.2 (8.7–100)	–

	No. in DUCA	Percentage in DUCA	Percentage in GCCG^A^	*P*-value (χ^2^/Fisher)
Patients with postoperative bowel obstruction	35	4.5	2.2	**0.005**
Postoperative bowel obstruction	12	34.3	–	–
Ileus	25	71.4	–	–
Severity score				
Grade I–II	18	48.6	–	–
Grade IIIa–IVb	16	43.2	–	–
Grade V	3	8.1	–	–
*Surgical procedure*				
Total gastrectomy	23	65.7	–	–
Subtotal gastrectomy	12	34.3	–	–
Patient requiring reinterventions (multiple options allowed)	24	68.6	–	–
Surgical	17	48.6	–	–
Endoscopic	11	31.4	–	–
Radiologic	6	17.1	–	–
Escalation in level of care	5	14.3	–	–
Failure to cure	3/35	8.6	–	–
Deceased patients with this complication	5	0.6	–	–
Postoperative hospitalization, days (median, range)	16 (4–105)	–	–	–
CCI (median, range)	33.7 (8.7–100)	–	–	–

	No. in DUCA	Percentage in DUCA	Percentage in GCCG^A^	*P*-value (*χ*^2^/Fisher)
Patients with other major complications requiring re-intervention	26	3.3	2.6	0.401
Other reoperations, not caused by bleeding, anastomotic leakage or interponate necrosis	3	10.7	–	–
Complication of jejunostomy	6	21.4	5.7	0.139
Evisceration	6	21.4	17.1	0.914
Diaphragmatic hernia	0	–	2.8	1
Other major complications	13	46.4	74.3	**0.045**
*Severity score*				
Grade I–II	0	–	0	1
Grade IIIa–IVb	20	71.4	91.4	0.081
Grade V	8	28.6	8.6	0.081
*Surgical procedure*				
Total gastrectomy	14	53.8	71.4	0.252
Subtotal gastrectomy	12	46.2	28.6	0.252
Patient requiring reinterventions (multiple options allowed)	22	84.6	100	0.060
Surgical	18	69.2	62.8	0.806
Endoscopic	6	23.1	8.6	0.225
Radiologic	6	23.1	–	–
Escalation in level of care	9	34.6	31.4	1
Failure to cure	8/26	30.8	8.6	0.058
Deceased patients with this complication	9	1.2	0.3	**0.031**
Postoperative hospitalization, days (median, range)	11 (2–105)	–	22.5 (4–83)	–
CCI (median, range)	39.7 (26.2–100)	–	39.7 (26.2–100)	–

^A^Complication according to the GCCG definitions: Baiocchi GL, Giacopuzzi S, Marrelli D, et al. International consensus on a complications list after gastrectomy for cancer. *Gastric Cancer* 2019

The overall anastomotic leakage rate was 6.6% in the Netherlands, compared to 4.5% in the GCCG cohort (*p* = 0.044) (Table 4). For total gastrectomy, these rates were 12.6% vs 7.2% (*p* = 0.005) and 1.2% vs 1.5% (*p* = 0.856) for subtotal gastrectomy, respectively. Patients with anastomotic leakage more often underwent an endoscopic reintervention in the Netherlands (61.5% vs 27.9%, *p* < 0.001), with surgical reintervention being the preferred reintervention in the GCCG cohort (42.6%). The median in-hospital stay for patients with anastomotic leakage was 19 days in the Netherlands compared to 32 days in the GCCG cohort.

### Additional complications in DUCA

Distinct complications in the DUCA that are not included in the GCCG definitions are shown in Table S3. When including these complications, the complication rate was 33.9% in the Netherlands. The most frequent complications were non-severe cardiac complications (CD I-II, 20.4%), other complications (CD grade I–II, 19.7%), acute delirium (12.9%), and urine retention resulting in prolonged catheter use (10.9%).

## Discussion

This study reported on postoperative complications after gastrectomy in the Netherlands according to the standardized complication definitions published by the GCCG [6]. The 30-day/in-hospital mortality rate was 4.1% and the postoperative complication rate was 28.5% per the GCCG definitions in the Netherlands. Morbidity and mortality rates are similar between the Netherlands and the GCCG cohort, consisting of patients from 27 European expert centers, despite significant variations in baseline characteristics.

The overall postoperative complication rate found in this study was lower than previously reported in the Netherlands. In 2017–2018, the complication rate after gastrectomy was 39.2%, compared to the 28.5% in this study (Table 2) [16]. As the DUCA registers additional complications, this decline is partly attributable to the stricter morbidity definitions of the GCCG. These additional complications include more esophagectomy-related complications and less life-threatening but relevant complications like delirium and urine retention (Table S2). As they are not gastrectomy-specific complications, they were not included in the GCCG definitions [6]. With all DUCA-registered complications included the complication rate in this study is 33.9%. This variation in morbidity rates with and without standardized complication definitions underlines the difficulties in comparing studies that lack these standardized definitions. Nonetheless, these results show a decline in the overall complication rate in the Netherlands over the past 5 years. In addition, the 30-day/in-hospital mortality and anastomotic leakage rates were slightly lower in the current study as well (4.1% vs 4.3% and 6.6% vs 7.5%, respectively). Factors that contributed to these declines might be further centralization, DUCA-initiated best practice sessions and/or Dutch surgeons gaining proficiency in the learning curve of minimally invasive gastrectomy [9, 10, 17, 18].

The DUCA and European GCCG cohorts differed extensively on baseline characteristics (Table 1). First, the GCCG cohort consisted of patients undergoing surgery in 2017–2018, whilst the DUCA cohort consisted of patients that underwent gastrectomy in 2020–2021. In the Netherlands, significantly more patients received neo-adjuvant treatment compared to the GCCG cohort. Also, the higher median number of positive lymph nodes in the Netherlands suggests a higher nodal stage, despite not being observed in clinical node diagnostics. Furthermore, the minimally invasive surgery rate was high in the Netherlands compared to a previous DUCA study in 2017–2018, the GCCG cohort and other European countries [11, 16, 19, 20]. The lymph node yield was found to be lower in Netherlands compared to the GCCG cohort (25 vs. 31). Various factors such as neo-adjuvant therapy, pathologist examination and procedural volume can influence lymph node yield [21]. The high laparoscopic gastrectomy rate might play a role as well, although this difference was not reported in the LOGICA-trial and a meta-analysis [22, 23]. Another explanation could be a more effective lymph node dissection performed by the GCCG expert centers compared to the average Dutch center. This difference in lymph node yield might indicate less surgical aggressiveness in the Netherlands. All these differences could have impacted the reported outcomes in both cohorts. Additionally, the GCCG cohort was composed of a selection of high-volume expert centers throughout Europe, as opposed to every center in the Netherlands, potentially causing selection bias when comparing nationwide results to those from specific centers. However, a comparison between the DUCA and the ‘expert’ GCCG cohort is warranted to identify areas for improvement in the Netherlands, as the GCCG cohort could be considered as the top end of gastric cancer surgery in Europe.

The postoperative morbidity according to GCCG definitions was similar between the Netherlands and European GCCG cohorts (28.5% vs 29.8%, Table 2), with similar 30-day mortality rates as well (3.7% vs. 3.6%). Recently, Schneider et al. defined outcome benchmarks for total and distal gastrectomy, providing centers with targets for expert care [24]. Although the DUCA and GCCG cohorts included all patient types, both exceeded European/American benchmarks in terms of morbidity and mortality, showing room for improvement in both cohorts.

In the Netherlands, non-surgical infections, pleural effusion requiring drainage, anastomotic leakage, other postoperative abnormal fluid collections requiring treatment, postoperative bowel obstruction and other major complications requiring reintervention were the most frequent complications, mostly similar to the GCCG cohort results despite large variation in baseline characteristics.

The anastomotic leakage rate was significantly higher in the Netherlands compared to the GCCG cohort (6.6% vs 4.5%, Table 4), especially for total gastrectomies (12.6% vs 7.2%), and other European studies [24–26]. Similar results were seen in the comparison of DUCA data to European results after esophagectomy [9]. This resulted in the implementation of quality improvement programs with annual best-practice meetings with esophagogastric surgeons to improve the anastomotic leakage rate after esophagectomy [9, 10]. Discussing the outcomes after gastrectomy in these meetings will be planned. However, even though anastomotic leakage percentages are slightly higher, a trend toward less invasive leakage treatment is seen in the Netherlands, through the higher endoscopic reintervention rate, and overall hospital stay is shorter with similar mortality rates in patients with anastomotic leakage, suggesting earlier identification and/or effective early treatment.

In this study, the overall in-hospital stay was shorter in the Netherlands compared to the European GCCG cohort (6 vs 9 days, Table 2). The higher minimally invasive gastrectomy rate could play a role in this difference. Although the LOGICA trial, comparing laparoscopic vs open gastrectomy, showed no significant difference in in-hospital stay, a recent study by Markar et al. investigating the dissemination of laparoscopic gastrectomy in the Netherlands before, during and after the LOGICA-trial reported a significant reduction in in-hospital stay in laparoscopic gastrectomy after LOGICA [18, 22]. A shorter in-hospital stay was also observed in patients with complications, especially after anastomotic leakage and other major complications requiring reinterventions (Table 4), potentially caused by more aggressive re-intervening in the Netherlands. Both the in-hospital stay in the DUCA and GCCG cohorts fall below the benchmark target of 11 days [24]. However, comparing in-hospital stay between countries is challenging due to differences in reimbursement structures across healthcare systems.

Both the surgical and endoscopic/radiologic reintervention rates are significantly higher compared to the GCCG cohort (Table 2). Especially the endoscopic/radiologic reintervention rate was increased, also seen in patients with anastomotic leakage and other postoperative abnormal fluid collections requiring treatment. This could have impacted the rates of severe complications in the Netherlands, as the Clavien–Dindo score is a treatment-related severity grading system [27]. Nonetheless, no significant difference was seen in the proportion of severe complications compared to the GCCG cohort. However, as this is a proportion, the higher number of minor complications lowers the proportion of severe complications. This is supported by the higher median CCI score per patient, used to include multiple postoperative complications, in the Netherlands (33.6 vs 26.7, Table 2), indicating more complications per patient and/or a higher complication severity [28]. In the Netherlands, an endoscopy is often used as a diagnostic instrument to detect anastomotic leakage, possibly resulting in a higher rate. Furthermore, a preference for less invasive procedures could be present in the Netherlands, as demonstrated by the high minimally invasive surgery rate. Whether aggressive re-intervening is more successful can be debated, as the failure-to-rescue rates were similar in this study, although it potentially leads to a shorter in-hospital stay after complications.

The factors causing the higher proportion of intra-operative unintended damage to major vessels and/or organs in this study compared to the GCCG cohort are unknown. Two important differences in baseline characteristics, the higher minimally invasive surgery and neo-adjuvant therapy rates, are not considered the cause as both have not been proven to increase intra-operative complications [22, 29–31]. Although the incidence and differences are small (14 vs 7, Table 3), further reduction of intra-operative complications should remain a goal for Dutch surgeons.

This study is the first national cohort study reporting on complications after gastrectomy using the standardized GCCG definitions. It shows comparable data, leading the way to a broader implementation of these definitions. Since these were published only recently (2019), only a few studies have been able to fully report complications accordingly [11, 26]. For esophageal cancer surgery, a review of the implementation of the ECCG complication definitions showed adoption in 48.6% of studies on esophagectomies, with only one study reporting the entire complication set, 5 years after the publication [9, 32]. Full implementation could reduce the heterogeneity in the reporting of complications and allow for more accurate comparisons of international studies.

This study has some limitations. Although the DUCA was amended to the GCCG definitions in 2020, minor differences in the definition of the existing complication variables, which could not be corrected for by the Clavien–Dindo score, remained (Table S1). This could have caused the significantly higher postoperative bowel obstruction rate in the Netherlands compared to the GCCG cohort. Also, the higher rate of Clavien–Dindo grade II complications and complications per patient in the Netherlands could be caused by a mildly stricter definition of complications already included in the DUCA before the GCCG definitions. Since the complete GCCG dataset was unavailable, it was not possible to conduct statistical analyses on the continuous outcome measures. Whilst the DUCA showed accurate data registry after verification of the data [33], no such check has been performed of the GASTRODATA database, possibly influencing results. However, missing data were mostly comparable between both cohorts (Table 1).

In conclusion, this study shows that morbidity and mortality rates after gastrectomy are comparable between the Netherlands and the GCCG expert centers, but both exceed the international benchmark for expert gastrectomy care. A decrease in the overall complication rate was seen in the Netherlands, partly attributable to the stricter complication definitions of the GCCG, indicating the difficulties in comparing studies lacking uniform complication definitions. Dutch surgeons appear more aggressive in re-intervening in postoperative complications than their European counterparts, most notably using endoscopic and radiologic reinterventions, possibly resulting in the shorter hospital stay in case of complications. Reporting postoperative complications after gastrectomy according to standardized GCCG definitions allows for the accurate comparison of studies and international benchmarking. Identifying international differences provides valuable targets for quality improvement of gastric cancer surgery worldwide.

### Supplementary Information

Below is the link to the electronic supplementary material.

Supplementary file1 (DOCX 26 kb)
