# Supplementary material for: Outcomes after gastrectomy according to the Gastrectomy Complications Consensus Group (GCCG) in the Dutch Upper GI Cancer Audit (DUCA)
Source: Gastric Cancer. 2024 Jun 28;27(5):1124–35. doi: 10.1007/s10120-024-01527-0 (PMC11335793; doi:10.1007/s10120-024-01527-0)
Supplement: Supplementary file 1 — Supplementary file1 (DOCX 26 kb) [file 10120_2024_1527_MOESM1_ESM.docx]

| **Table S1:** Overview of definitions in GCCG and DUCA |  |
| --- | --- |
| **GCCG definitions^A^** | **DUCA matched definitions** |
| **Intraoperative** |  |
| **1. Unintended intraoperative damage to major vessels and/or organs requiring reconstruction or resection** (complete section of or major damage to hepatic/splenic artery, portal/cava vein, bile duct requiring reconstruction; damage to spleen requiring splenectomy; unplanned bowel resection / section of pancreas requiring resection (not for oncological reasons)) | 1. Damage to vessel resulting in bleeding requiring transfusion; damage to bowel requiring resection or reconstruction; damage to spleen requiring splenectomy; damage to pancreas |
| **2. Intraoperative bleeding requiring urgent treatment** | 2. Exact |
| **3. Unexpected medical conditions interrupting or changing the planned procedure** | 3. Not present in DUCA |
|  |  |
| **Postoperative general** |  |
| **4. Stroke causing patient’s permanent deficit** | 4. Exact |
| **5. Need for CPR** (regardless of underlying cause) | 5. Cardiac arrest requiring CPR |
| **6. Myocardial infarction** (patient needs to be transferred to CCU/ICU/other critical care facility, irrespective of treatment) | 6. Myocardial infarction & CD ≥IVa |
| **7. Cardiac dysrhythmia requiring invasive treatment** (atrial or ventricular) | 7. Exact |
| **8. Acute myocardial failure with acute pulmonary edema** (pulmonary edema clinically detected or lowered ejection fraction of >50%) | 8. Exact |
| **9. Pulmonary embolism** (symptoms confirmed by urgent CT scan) | 9. Exact |
| **10. Respiratory failure requiring reintubation** | 10. Exact |
| **11. Need for tracheostomy** | 11. Exact |
| **12. Pleural effusion requiring drainage** | 12. Exact |
| **13. Pneumothorax requiring treatment** | 13. Exact |
| **14. Need for prolonged intubation** (> 24 h after surgery) | 14. Exact |
| **15. Acute liver dysfunction** (Child–Pugh score > 8 for 48+ hours) | 15. Exact |
| **16. Acute renal insufficiency/renal failure requiring CVVH or dialysis** (postoperative creatinine twice its preoperative value/renal failure requiring CVVH or dialysis) | 16. Acute renal insufficiency with 100% increase of baseline creatinine; acute renal failure requiring dialysis. |
| **17. Non-surgical infections** (includes gastrointestinal, respiratory, renal/urinary and other infections) | 17. Pneumonia, pericarditis, Clostridium Difficile infection, urinary tract infection, peripheral thrombophlebitis, central line infection, generalized sepsis, other infections requiring antibiotics |
| **Postoperative surgical** |  |
| **18. Postoperative bleeding requiring both urgent transfusion and invasive treatment** (endovascular or endoscopic or surgical) | 18. Postoperative bleeding requiring transfusion and/or reintervention (not being gastrointestinal bleeding) |
| **19. Postoperative bowel obstruction** (clinical/radiological signs of obstruction, inability to enteral feed, longer need for NG suction) | 19. Ileus (dysfunction of small bowel requiring delay of enteral feeding); small bowel obstruction |
| **20. Postoperative bowel perforation or necrosis requiring surgical treatment** | 20. Necrosis of gastric tube, colon-/jejunum interponate; damage of bowel |
| **21. Duodenal leak** (irrespective of presentation, method of identification, clinical consequences, and treatment; abscess close to duodenal stump) | 21. Exact |
| **22. Anastomotic leak** (irrespective of presentation, method of identification, clinical consequences, and treatment; abscess close to anastomosis) | 22. Exact |
| **23. Postoperative pancreatic fistula** (drain output with amylase levels >3 times the upper limit of institutional normal serum amylase activity) | 23. Exact |
| **24. Postoperative pancreatitis diagnosed both clinically and radiologically** (postoperative serum amylases/lipases >3 times normal value and radiological signs of pancreatitis) | 24. Serum amylase >3 times the upper limit |
| **25. Other postoperative abnormal fluid from drainage and/or abdominal collections without gastrointestinal leak(s) preventing drainage removal and/or requiring treatment** (postoperative biliary drain, chylous ascites, and other abnormal fluids requiring (preventive) drainage or prolonged existing drainage, as well as abdominal collections requiring invasive treatment) | 25. Postoperative biliary drain, chylous ascites, other abnormal fluids requiring (preventive) drainage or prolonged existing drainage; chyle leakage; intra-thoracic / intra-abdominal abscess requiring drainage (radiological or surgical) |
| **26. Delayed gastric emptying by 10th postoperative day** (failure to tolerate oral intake in absence of bowel obstruction requiring reintervention or delayed discharge) | 26. Exact |
| **27. Other major complications requiring re-intervention or other invasive procedures** (includes evisceration, diaphragmatic hernia, feeding jejunostomy-related complications, etc., which require reintervention or other invasive procedures | 27. Other reoperations, not caused by bleeding, anastomotic leakage or interponate necrosis; feeding jejunostomy-related complication; fascia-dehiscence, platzbauch or acute hernia; acute diaphragmatic hernia; other complications (all above CD≥IIIa) |
| A. Complication definititions according to the GCCG definitions: Baiocchi GL, Giacopuzzi S, Marrelli D, et al. International consensus on a complications list after gastrectomy for cancer. *Gastric Cancer* 2019. | |

| **Table S2**: Characteristics of all reinterventions in the DUCA cohort. | | |
| --- | --- | --- |
|  | No. | Percentage |
| Patients with a reintervention | 144 | 18.4 |
| Re-operation | 84 | 10.7 |
| Endoscopic | 58 | 7.4 |
| Radiologic | 41 | 5.2 |
| Reason for reintervention |  |  |
| Anastomotic leakage | 48 | 33.3 |
| Chyle leakage | 0 | 0 |
| Postoperative bleeding | 12 | 8.3 |
| Evisceration | 5 | 3.5 |
| Intra-abdominal abscess | 10 | 6.9 |
| Damage of small bowel | 7 | 4.9 |
| Necrosis of gastric conduit/colon-/jejunum interponate | 1 | 0.7 |
| Pancreatitis | 1 | 0.7 |
| Epidural-related complication | 0 | 0 |
| Jejunostomy-related complication | 5 | 3.5 |
| No complication identified during procedure | 10 | 6.9 |
| Other | 45 | 31.3 |
| Unknown | 0 | 0 |

| **Table S3:** Incidence and grading of all postoperative complications not in the GCCG^A^ definitions. | | | |
| --- | --- | --- | --- |
| Total patient episodes = 782 | Number of adverse events | % of adverse events | Clavien-Dindo score (median) |
| Patients developing at least 1 complication (all included) | 265 | 33.9 | II |
| Patients developing a complication not in GCCG definitions | 104 | 14.1 | II |
| **Intraoperative** |  |  |  |
| Other intraoperative complications (no definition) | 9 | 6.1 | - |
| **Postoperative general** |  |  |  |
| Atelectasis caused by mucus plug resulting in bronchoscopy | 1 | 0.7 | IVa |
| Acute aspiration | 10 | 6.8 | IVa |
| Acute Respiratory Distress Syndrome (ARDS)^B^ | 6 | 4.1 | IVb |
| Persisting air leakage requiring thorax drain | 0 | 0 |  |
| Urine retention resulting in re-insertion of catheter, prolonged in-hospital stay or discharge with catheter | 16 | 10.9 | I |
| Deep venous thrombosis | 1 | 0.7 | II |
| Acute delirium | 19 | 12.9 | II |
| Delirium tremens | 2 | 1.4 | II |
| Other neurological complications (no definition) | 0 | 0 | - |
| Multiple Organ Dysfunction Syndrome (MODS) | 3 | 2.0 | V |
| Cardiac complications CD grade I-II | 30 | 20.4 | II |
| Other complications (CD grade I-II) | 29 | 19.7 | II |
| **Postoperative surgical** |  |  |  |
| Pyloromyotomy / pyloroplasty-related complication | 0 | 0 | - |
| Damage of recurrent nerve | 0 | 0 | - |
| Wound infection | 12 | 8.2 | II |
| Wound dehiscence | 4 | 2.7 | I |
| Epidural-related complication | 1 | 0.7 | I |
| Other surgical complications (CD grade I-II) | 4 | 2.7 | II |
| Total | 147 |  |  |
|  | **Median** | **Range** |  |
| Comprehensive Complications Index (CCI) all complications | 33.5 | (8.7 – 100) |  |
| Comprehensive Complications Index (CCI) GCCG complications | 33.5 | (8.7 – 100) |  |
| Postoperative hospitalization, days all complications | 11 | (2 - 105) |  |
| Postoperative hospitalization, days GCCG compilations | 13.5 | (2 - 105) |  |
| Number of days on ICU | 0 | (0 - 47) |  |
| A. GCCG definitions: Baiocchi GL, Giacopuzzi S, Marrelli D, et al. International consensus on a complications list after gastrectomy for cancer. *Gastric Cancer* 2019.  B. Berlin definition | | | |
